# 

**Published:** 1897-01-16

**Authors:** 


### EARLY EDITION.

**Notices of Births, Deaths, and Marriages** are inserted in this column at a charge of 2s. 6d. for an announcement not exceeding four lines.

### NOTICE TO CORRESPONDENTS.

All MS., Letters, Books for Review, and other matters intended for the Editor should be addressed THE EDITOR, "THE HOSPITAL," THE HOSPITAL BUILDING, 28 & 29, SOUTHAMPTON STREET, STRAND, W.C.

The Editor cannot undertake to return rejected MS., even when accompanied by stamped directed envelope.

**Notice to Advertisers and Subscribers.**

All Advertisements, Orders for Copies of THE HOSPITAL, and Busines Communications should be addressed to **THE MANAGER, (Not to The Editor),** THE HOSPITAL, The Hospital Building, 28 & 29, Southampton Street, Strand, London, W.C.

The Cost of Subscription, post free, is as follows:—Per week, 2½d.; per quarter, 2s. 8d.; per half-year, 5s. 3d.; per annum, 10s. 6d. Foreign:—Per annum, 15s. 2d.; payable, in all cases, in advance.

**NOTICE.—Vol. XX. of "The Hospital" (April to September, 1896), in handsome cloth binding, is now on sale at the Office of this Journal, price 7s. 6d. Cases for binding the Numbers contained in this Volume. 1s. 6d. Index to Vol. XX., 2½d., post free. The Monthly Part for January is now ready. Price 6d., by post 8d.**

### The Student of Medicine.

### "DISGUYSED" MINSTRELS AT ST. GEORGE'S HALL.

An entertainment was given by the students of Guy's Hospital in St. George's Hall on the evening of January 11th, before H.R.H. Duke of Cambridge and a large audience. The proceedings opened with an overture by the student's orchestra, who were throughout ably conducted by Mr. Balfour D. Adamson. The first part of the programme consisted in a Christy-Minstrel performance by the whole of the "disguysed" minstrel troupe. There were numerous songs freely interspersed with apparently spontaneous witticisms on the part of the bones and the tambourines. It would be hard to select the best of all the songs rendered, but the first encore was accorded to Mr. A. A. Miller's "I've Got the Oopeizootic"—and quite deservedly so. Mr. T. L. Jackson was well worthy of remark in "Dinah, dear, Dinah"; and immediately following this, Mr. J. E. Collins, though apparently suffering from a cold, was really comic in "Wait till de Sun am Hot upon de Head." Mr. Cazenove kept the audience laughing in "Rhoda,' and earned a well-deserved encore. The next item on the programme was "Whisper, and I shall Hear," sung with great feeling by Mr. L. E. C. Handson, who was at once called upon to sing again. The great triumph of the first part of the entertainment was, however, achieved by Mr. A. Hay, who posed as the "Dandy Coloured Coon," and was really excellent, his acting being as good as his rendering of the song, which he performed with great verve and accuracy. Mr. G. Lewis's song, "It was a Liner," with chorus by the troupe, was the concluding item. A word of praise is due to the business-like way in which the "Disguysed" Minstrels fulfilled their *rôle*, winning as they did a large share of appreciation from their audience. During the *entr'acte* the orchestra were to the fore in their selections from the "Geisha." When the curtain rose on Part II. Mr. P. H. Lulham gave a musical sketch. To the experienced observer it would seem that Mr. Lulham had never been meant for any thing else but a successor to the late Mr. Corney Grain, who was always in evidence on the same platform at this season of the year only a comparatively short time ago. His description of the pantomime was in exceedingly good taste and very amusing. Mr. Collins contributed to Part II. with his "Inventions," which are decidedly novel, even if they aspire to nothing else. Next came a dance by Mr. C. Shepherd, which gained an encore, and justly so, too, for it was distinctly clever, Mr. Cazenove's music supplying an able accompaniment. Last, but by no means least, Mr. Stanley W. Cock drew some excellent lightning sketches of celebrities, which were on the whole quite the cleverest part of the whole evening's entertainment. In conclusion, Messrs. Cazenove, Miller, and Hay were seen to distinct advantage in a so-called "tragedy," "Hamlet, or the Dress Rehearsal!" This little piece was a very fitting conclusion to a thoroughly good evening's entertainment, and we may heartily congratulate the students of Guy's Hospital who took part, not only on the meritorious success of their first West-end performance, but also on the worthiness of their object and the substantial addition to the funds of Guy's Hospital which must accrue, judging from the crowded audience. The "Disguysed" Minstrels are well worth seeing, so we may add that they give evening performances at the Brixton Hall and Crystal Palace Theatre on January 25th and February 23rd respectively.

### APPOINTMENTS.

B. Anningson, M.D.Camb., reappointed Medical Officer of Health to the Cambridge Town Council. S. Beatty, M.B., C.M.Edin., appointed Medical Officer of Health to the Moulin Parish Council. W. E. Bennett, F.R.C.S.Eng., appointed Resident Surgical Officer to the General Hospital, Birmingham. Dr. Davey, appointed Medical Officer for the Second Division of the St. James's District of the Dover Union. D. M. Forbes, L.R.C.P.Edin., L.F.P.S.Glasg., appointed Medical Officer of Health to the Eastwood Urban District Council. A. Hodge, L.R.C.P.. M.R.C S., appointed Resident Medical Officer to the Manchester Hospital for Consumption and Diseases of the Throat. Mr. M. Jones, appointed Medical Officer for the Glyn Ceiriog and the Llangollen District of the Corwen Union. R. Legge, M.D., appointed Medical Superintendent to the Derby County Asylum. D. Lloyd, M.B., C.M.Glasg, appointed Medical Officer for the Llanrhaiadr District of the Ruthin Union. J. G. Mapleton, M.B., appointed Medical Officer to the Cranbrook Union. H. W. Pepper, M.R.C.S, L.R.C.P., appointed Assistant Medical Officer to the Workhouse Infirmary, Birmingham. W. A. Smyth, L.R.C.P., L R C S Edin., appointed Medical Officer and Public Vaccinator for the Ashstead District of the Epsom Union. G. H. Temple, M.B., C.M Edin., appointed Medical Officer for the Weston-super-Mare District of the Axbridge Union. A. Thorp, M.R.C.S.Eng., L.R.C.P.Lond., appointed Medical Officer of Health to the Urban Sanitary District of New Mills, Yorks.

